# Diversity and evolution of myxozoan minicollagens and nematogalectins

**DOI:** 10.1186/s12862-014-0205-0

**Published:** 2014-09-29

**Authors:** Erez Shpirer, E Sally Chang, Arik Diamant, Nimrod Rubinstein, Paulyn Cartwright, Dorothée Huchon

**Affiliations:** Department of Zoology, Tel Aviv University, Tel Aviv, Israel; Department of Ecology and Evolutionary Biology, University of Kansas, Lawrence, USA; National Center for Mariculture, Israel Oceanographic and Limnological Research, Eilat, Israel; Department of Molecular and Cellular Biology, Harvard University, Cambridge, USA

**Keywords:** Polar capsules, Nematocysts, Phylum-restricted gene, Cnidaria, Myxozoa, *Polypodium*, Molecular evolution

## Abstract

**Background:**

Myxozoa are a diverse group of metazoan parasites with a very simple organization, which has for decades eluded their evolutionary origin. Their most prominent and characteristic feature is the polar capsule: a complex intracellular structure of the myxozoan spore, which plays a role in host infection. Striking morphological similarities have been found between myxozoan polar capsules and nematocysts, the stinging structures of cnidarians (corals, sea anemones and jellyfish) leading to the suggestion that Myxozoa and Cnidaria share a more recent common ancestry. This hypothesis has recently been supported by phylogenomic evidence and by the identification of a nematocyst specific minicollagen gene in the myxozoan *Tetracapsuloides bryosalmonae*. Here we searched genomes and transcriptomes of several myxozoan taxa for the presence of additional cnidarian specific genes and characterized these genes within a phylogenetic context.

**Results:**

Illumina assemblies of transcriptome or genome data of three myxozoan species (*Enteromyxum leei*, *Kudoa iwatai*, and *Sphaeromyxa zaharoni*) and of the enigmatic cnidarian parasite *Polypodium hydriforme* (Polypodiozoa) were mined using tBlastn searches with nematocyst-specific proteins as queries. Several orthologs of nematogalectins and minicollagens were identified. Our phylogenetic analyses indicate that myxozoans possess three distinct minicollagens. We found that the cnidarian repertoire of nematogalectins is more complex than previously thought and we identified additional members of the nematogalectin family. Cnidarians were found to possess four nematogalectin/ nematogalectin-related genes, while in myxozoans only three genes could be identified.

**Conclusions:**

Our results demonstrate that myxozoans possess a diverse array of genes that are taxonomically restricted to Cnidaria. Characterization of these genes provide compelling evidence that polar capsules and nematocysts are homologous structures and that myxozoans are highly degenerate cnidarians. The diversity of minicollagens was higher than previously thought, with the presence of three minicollagen genes in myxozoans. Our phylogenetic results suggest that the different myxozoan sequences are the results of ancient divergences within Cnidaria and not of recent specializations of the polar capsule. For both minicollagen and nematogalectin, our results show that myxozoans possess less gene copies than their cnidarian counter parts, suggesting that the polar capsule gene repertoire was simplified with their reduced body plan.

**Electronic supplementary material:**

The online version of this article (doi:10.1186/s12862-014-0205-0) contains supplementary material, which is available to authorized users.

## Background

Myxozoa is a group of endoparasites comprising over 2,000 described species [1]. Members of this group are pathogens of salmonid, sparids and other economically important aquacultural fish. In particular, myxozoan infections are responsible for whirling disease, proliferative kidney disease, ceratomyxosis, enteromyxosis and kudoasis [2,3]. There is no known effective treatment against myxozoan infections, and as a result they can cause severe economic losses in fish farms [4,5]. Nevertheless, the vast majority of species produce benign infections that are typically asymptomatic.

The polar capsule is a highly complex intracellular structure found in myxozoan spores. Polar capsules play a critical role in myxozoan infection, as the everted tubule of the polar capsule is what presumably anchors the myxozoan spore to its host [6]. Despite the remarkable complexity and vital role polar capsules play in myxozoan parasitism, little is known about the structure and function of this organelle that characterizes this diverse group of microscopic parasites.

Myxozoans were originally classified as protists, but current molecular [7,8] and morphological [9,10] evidence supports their phylogenetic placement as metazoans. Their position among metazoans is debated (reviewed in [11]), however recent data suggest that they are highly degenerate members of Cnidaria (e.g., sea anemones, corals, hydras and jellyfish) [8], and possibly the sister taxon to *Polypodium hydriforme*, an enigmatic cnidarian parasite of the oocytes of sturgeon and paddlefish (Acipenseridae) [7,12,13].

The long appreciated observation that the myxozoan polar capsule bears remarkable similarity to the nematocyst, the stinging structure in cnidarians, gave rise to the hypothesis that myxozoans are in fact cnidarians [7,9,14]. Both myxozoan polar capsules and cnidarian nematocysts consist of a capsule whose wall is continuous with a coiled tubule that everts from its apical end. The apical opening of the polar capsule is thought to be covered by a hinged cap (operculum), a structure characteristic of nematocysts from medusozoan cnidarians (e.g., jellyfish, Hydra and hydroids) and absent in anthozoans (e.g., corals, sea anemones and sea pens)[15]. The morphological affinities between polar capsules and nematocysts have been supported by the finding that the myxozoan *Tetracapsuloides bryosalmonae* possesses a nematocyst-specific gene called minicollagen [16].

Although little is known about the molecular basis of myxozoan polar capsules, extensive research has been conducted on the molecular composition of cnidarian nematocysts, especially in the model system *Hydra* [17]. Several genes expressed in the nematocyst are purportedly unique to the phylum Cnidaria [18,19]. Among the 410 nematocyst proteins of *Hydra* [19], four gene families have been well characterized; the minicollagen family [20–22], nematocyst outer wall antigen (NOWA) [23,24], spinalin [25,26] and the nematogalectin family [27].

**Figure 1:**
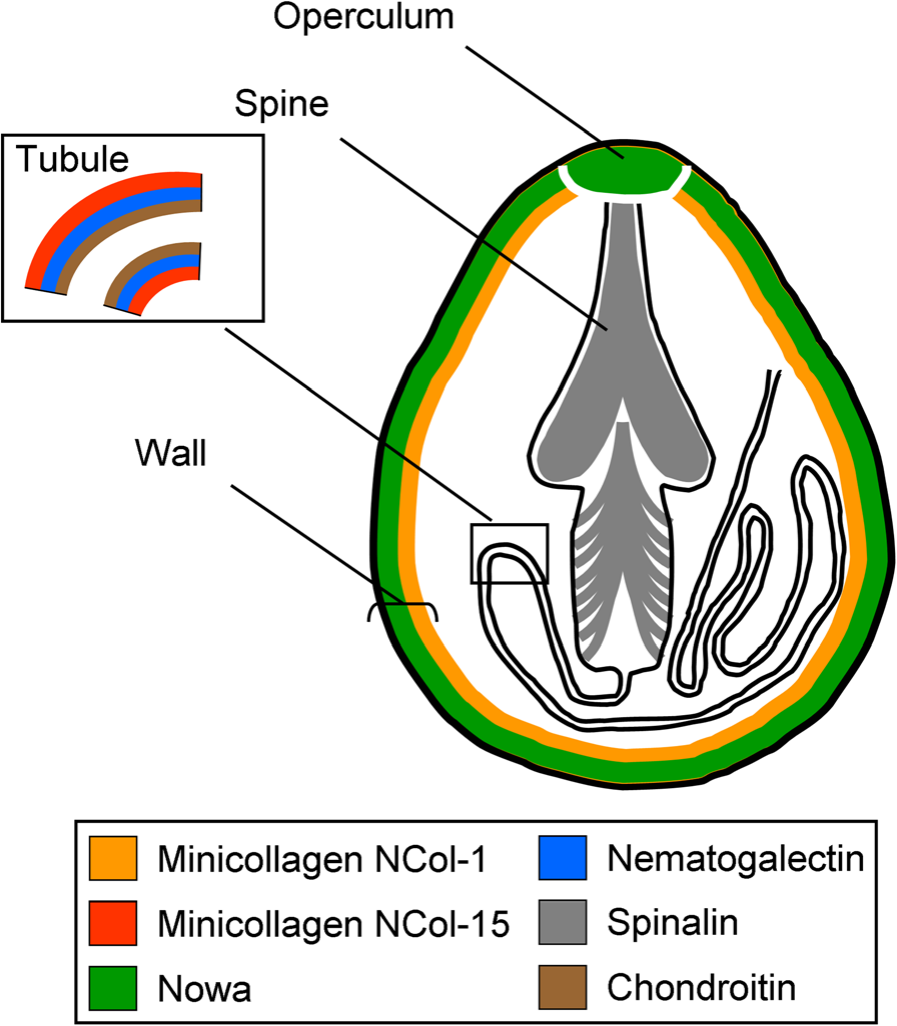
**Schematic drawing of chondroitin**, **nematogalectin,** **NCol-** **15,** **NCol-** **1,** **NOWA**, **and spinalin antigen distribution in a mature nematocyst (stenotele) of Hydra.** (based on Engel et al. [23] and Adamczyk et al. [28]).

Minicollagens are the primary structural components of nematocysts [20,21]. In *Hydra*, different minicollagens are expressed in different parts of the nematocyst. For example, the minicollagen NCol-15 is localized to the tubule, while the minicollagen NCol-1 is localized in the capsule wall [22] (Figure 1). Interestingly, hydrozoans, which possess the largest diversity of nematocyst types, have been found to encode the largest number of minicollagen genes. It has thus been suggested that the expansion of this gene family, through gene duplication and sequence divergence, has been largely responsible for the complexity of this structure and diversity of nematocyst types found throughout the phylum [21]. A single minicollagen gene has been identified in the myxozoan *Tetracapsuloides bryosalmonae* [16].

Another gene characterized to be specific to nematocysts is NOWA (nematocyst outer wall antigen) which interlinks with the minicollagens to produce a scaffold strong enough to withstand the pressure of discharge [23]. The spinalin protein is present in the spines and operculum of *Hydra* nematocysts [25].

Finally, nematogalectins are a family of genes among which some members have been found to interact with NCol-15 and a non-sulfated form of chondroitin to form the tubule wall [22,27], (Figure 1). Hwang et al. [27] described three members of the nematogalectin family in cnidarians. The nematogalectin genes were called nematogalectin A, nematogalectin B and nematogalectin-related. Hydrozoans were found to possess all three nematogalectins genes while anthozoans possessed only nematogalectin B and nematogalectin-related genes.

We searched for orthologs of these four cnidarian specific gene families in newly generated genome and/or transcriptome assemblies from three different myxozoan species as well as the putative sister taxon to Myxozoa, *Polypodium hydriforme* (Table 1) [7,13]. Newly discovered genes were characterized in a phylogenetic context in order to investigate the origin and evolution of these cnidarian-restricted gene families.

**Table 1:** **Summary of genomic resources utilized and cnidarian specific genes recovered**

**Taxon**	**Classification**	**Assemblies**	**Number of cnidarian specific genes recovered**				**Nematocyst types**
			**Nematogalectins**	**Minicollagens**	**NOWA**	**Spinalin**	
*Kudoa iwatai* Egusa & Shiomitsu, 1983	Myxozoa: Myxosporea	GEN/TRAN	3	3	0	0	Polar capsule
*Sphaeromyxa zaharoni* Diamant, Whipps & Kent, 2004	Myxozoa: Myxosporea	GEN	3	3	0	0	Polar capsule
*Enteromyxum leei* (Diamant, Lom & Dykova, 1994)	Myxozoa: Myxosporea	GEN	3	3	0	0	Polar capsule
*Buddenbrockia plumatellae* Schröder, 1910 / *Tetracapsuloides bryosalmonae* (Canning, Okamura & Curry, 1996)	Myxozoa: Malacosporea	EST	0^1^	3	0	0	Polar capsule
*Polypodium hydriforme* Ussov, 1885	Polypodioza	TRAN	4	11	0	0	Atrichous isorhiza and holotrichous isorhizas (4 types) [29]
*Hydra vulgaris* Pallas, 1766	Hydrozoa	GEN	4	21^2^	1	1	Desmoneme, atrichous isorhiza, holotrichous isorhiza; and stenotele [30,31]
*Nematostella vectensis* Stephenson, 1935	Anthozoa	GEN	4	5	0	0	Basitrichous haplonema, microbasic mastigophore, spirocyst [32]

^1^The absence of nematogalectin transcript may be due to the quality of the transcriptome assembly.

^2^A total of 21 minicollagen transcripts have been described for *Hydra*, however only 16 follow the canonical minicollagen structure.

*De novo* genomic and transcriptomic assemblies generated in this study are shown in bold. GEN = genomic assemblies, EST= expressed sequence tags, TRAN = transcriptomic assemblies.

## Results

### Mining myxozoan transcriptomes and genome assemblies for nematocyst genes

Among the four cnidarian specific protein families considered (minicollagens, NOWA, spinalin and nematogalectin), we were able to identify members of the minicollagen and nematogalectin gene families in three of the four myxozoan species (Table 1). Three different minicollagen sequences were identified in the transcriptome of *Kudoa iwatai*. We then verified that these transcripts correspond to three different genes by searching the corresponding genes in the DNA assembly. In addition, orthologous copies of these three genes were found in genomic assemblies for the other two myxozoan species investigated.

Searching publicly available EST data recovered two additional minicollagen sequences in *Buddenbrockia plumatellae*, a myxozoan species which, together with *T. bryosalmonae*, belong to the class Malacosporea [33,34]. A greater diversity of minicollagens was found in the transcriptome of the cnidarian parasite, *Polypodium hydriforme*, with the identification of 11 unique minicollagen transcripts (Table 1).

Minicollagen sequences of *Hydra* start with a signal peptide (this sequence is different for each minicollagen) [20,22]. We verified that the presence of a signal peptide could be predicted for all myxozoan and *Polypodium* minicollagen sequences. This also suggests that the 5′-end of the CDS has been correctly identified.

Our searches for nematogalectin orthologs led us to identify three genes which belong to this family in myxozoans, and four in *Polypodium* (Table 1). Our reciprocal Blast searches (see Methods) lead us to discover that cnidarians contain additional members of this family that were not described by Hwang et al. [27]. Specifically, we found a novel clade of nematogalectin proteins here called nematogalectin-C, which was found to be present in anthozoans, medusozoans, *P. hydriforme* and myxozoans. Unlike other nematogalectins, sequences within clade C are characterized by a long variable N-terminal end. This long N-terminal region could only be completely characterized for a few species with transcriptomic data of high quality (i.e., *N. vectensis*, *H. vulgaris*, *A. digitifera*, *P. hydriforme*). Like other nematogalectins, all complete nematogalectin-C sequences (except the sequence of *Hydra*) were found to begin with a signal peptide. The signal peptide region could not be identified in the predicted nematogalectin-C sequences of the sampled myxozoans, and the *Kudoa* and *Enteromyxum* sequences do not start with a methionine. This suggests that these sequences are incomplete. We also discovered additional anthozoan nematogalectin sequences. These sequences were found to be more similar to nematogalectin A and B sequences than to nematogalectin C or nematogalectin-related sequences (Additional files 1 and 2). They were thus assumed to be the missing anthozoan nematogalectin A copies that were not identified by Hwang et al. [27]. The sequences were thus named *Nematostella vectensis* A, *Anemonia viridis* A, *Metridium senile* A, *Acropora digitifera* A, *Acropora millepora* A. All these new sequences were predicted to begin with a signal peptide.

Our analyses thus indicate that cnidarians (including *P. hydriforme*) possess four different nematogalectin genes while myxozoans have only three (nematogalectin A, nematogalectin C, and nematogalectin-related). We were unable to identify nematogalectin sequences in the EST of *B. plumatellae* and *T. bryosalmonae* available in NCBI.

Our Blast searches did not identify any putative orthologs for the NOWA and spinalin proteins in the myxozoan genomes or in the *Polypodium* transcriptome.

### Minicollagen phylogenetic relationships and protein structure

**Figure 2:**
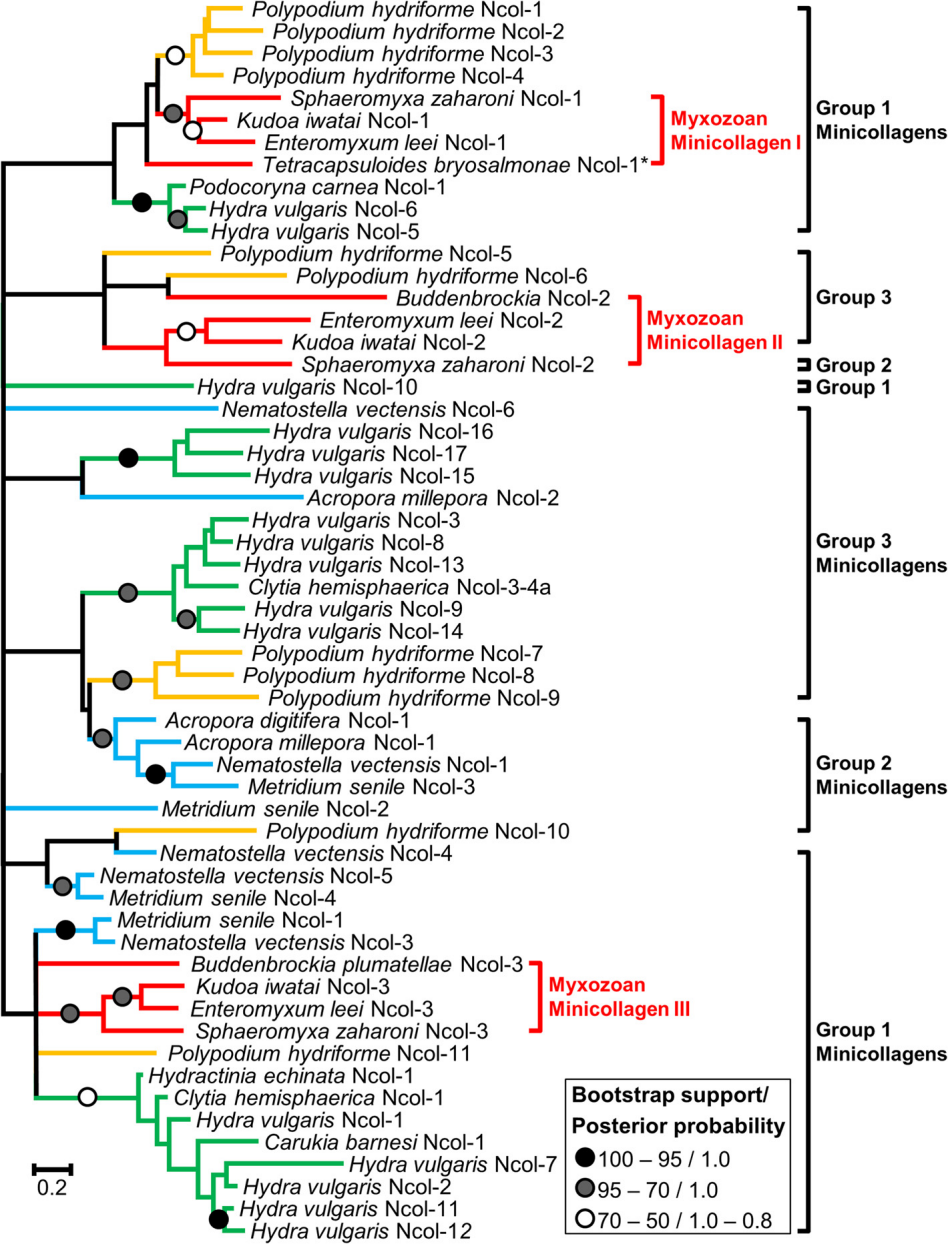
**ML phylogenetic tree reconstructed based on the minicollagen dataset.** Circles indicate node supports. Black circles: 100 ≥ BP ≥ 95, PP = 1.0. Gray circles: 95 > BP ≥ 70, PP = 1.0. White circles: 70 > BP ≥ 50, 1.0 ≥ PP ≥ 0.8. Nodes with a bootstrap support below 20% were collapsed. Blue, green, orange and red branches represent Anthozoa, Medusozoa, Polypodioza and Myxozoa respectively. Group 1 minicollagens are characterized by N-terminal and C-terminal CRDs which follow the pattern CXXXCXXXCXXXCXXXCC. Group 2 is characterized by N-terminal CRDs similar to that of group 1 minicollagens but with variable and different C-terminal CRD patterns. Finally, group 3 is characterized by variable CRD patterns both at the N-terminal and C-terminal [21]. * indicate that *Tetracapsuloides* Ncol-1 does not have the canonical CRD structure (cf. [16]).

Since minicollagens are encoded by short and fast evolving sequences, most branches within the tree are not well-supported (Maximum likelihood [ML] bootstrap percentage BP < 50%, Bayesian posterior probability PP < 0.7) (Figure 2), precluding reconstruction of the order of the duplication events. In addition, it is possible that the number of minicollagen genes is underestimated for species for which only EST data are available (e.g., *Clytia hemisphaerica*, *Carukia barnesi*).

The myxozoan minicollagens were found to belong to three different clades, which are not well-supported (BP = 65/19/-, PP = 1.0/-/1.0, for clades I, II and III respectively). The minicollagens from *Polypodium* cluster with myxozoan minicollagens in clades I and II (Figure 2). The myxozoan minicollagen II sequences (Figure 2, *Sphaeromyxa zaharoni* Ncol-2, *Kudoa iwatai* Ncol-2, *Enteromyxum leei* Ncol-2, and *Buddenbrockia plumatellae* Ncol-2) present an unusual structure with a C-terminal region which contains three repeats of the “poly-Proline - CRD” region (Additional file 1). The two *Polypodium* sequences related to this clade (i.e., *Polypodium hydriforme* Ncol-5 and *Polypodium hydriforme* Ncol-6) possess two repeats of the “poly-Proline - CRD” region in their C-terminal region and one (Ncol-6) or two (Ncol-5) CRD repeats in their N-terminal region (Additional file 1).

**Figure 3:**
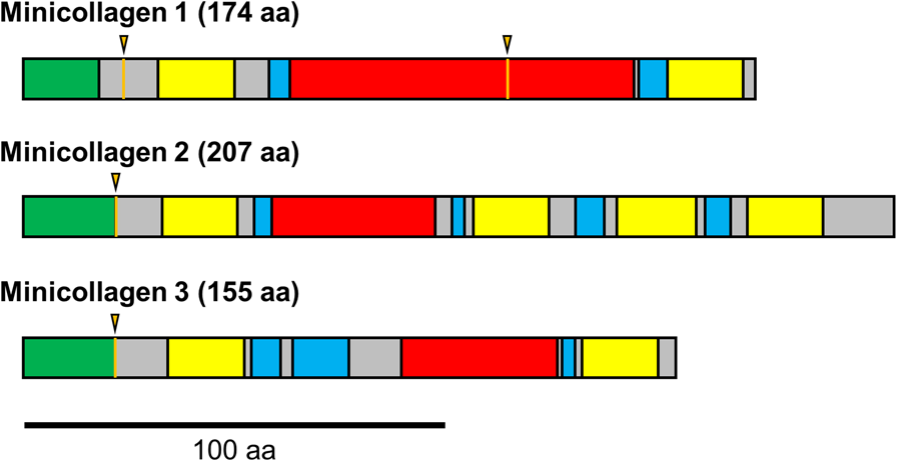
**Schematic drawing of** ***Kudoa iwatai*** **minicollagens.** Green: signal peptide; yellow: cysteine rich domain; blue: Poly-proline domain; red: collagen-like domain; orange line and black triangle: intron-exon boundary.

Comparison of the RNA transcripts with the DNA contigs in *Kudoa iwatai*, indicate that minicollagen I possesses two introns while minicollagens II and III possess only a single intron (Figure 3; Additional file 1). The same number of introns was also found for *Sphaeromyxa* and *Enteromyxum* with Augustus [35] intron/exon structure prediction.

The Trinity *de novo* assembler [36] divides its predicted contigs into components, where contigs belonging to the same component are similar to different isoforms of the same gene. Nevertheless, we could not find any evidence of alternative splicing in *Kudoa* since each minicollagen gene matched to a single RNA contig. In addition, each protein sequence in the *Polypodium* assembly was represented by two contigs, which differ by only a few base pairs on the third codon position. Since no evidence of alternative splicing was found among the *Polypodium* contigs and since we extracted RNA from several individuals (see Methods), these different contigs most likely represent allelic variability.

### Nematogalectin phylogenetic relationships and protein structure

**Figure 4:**
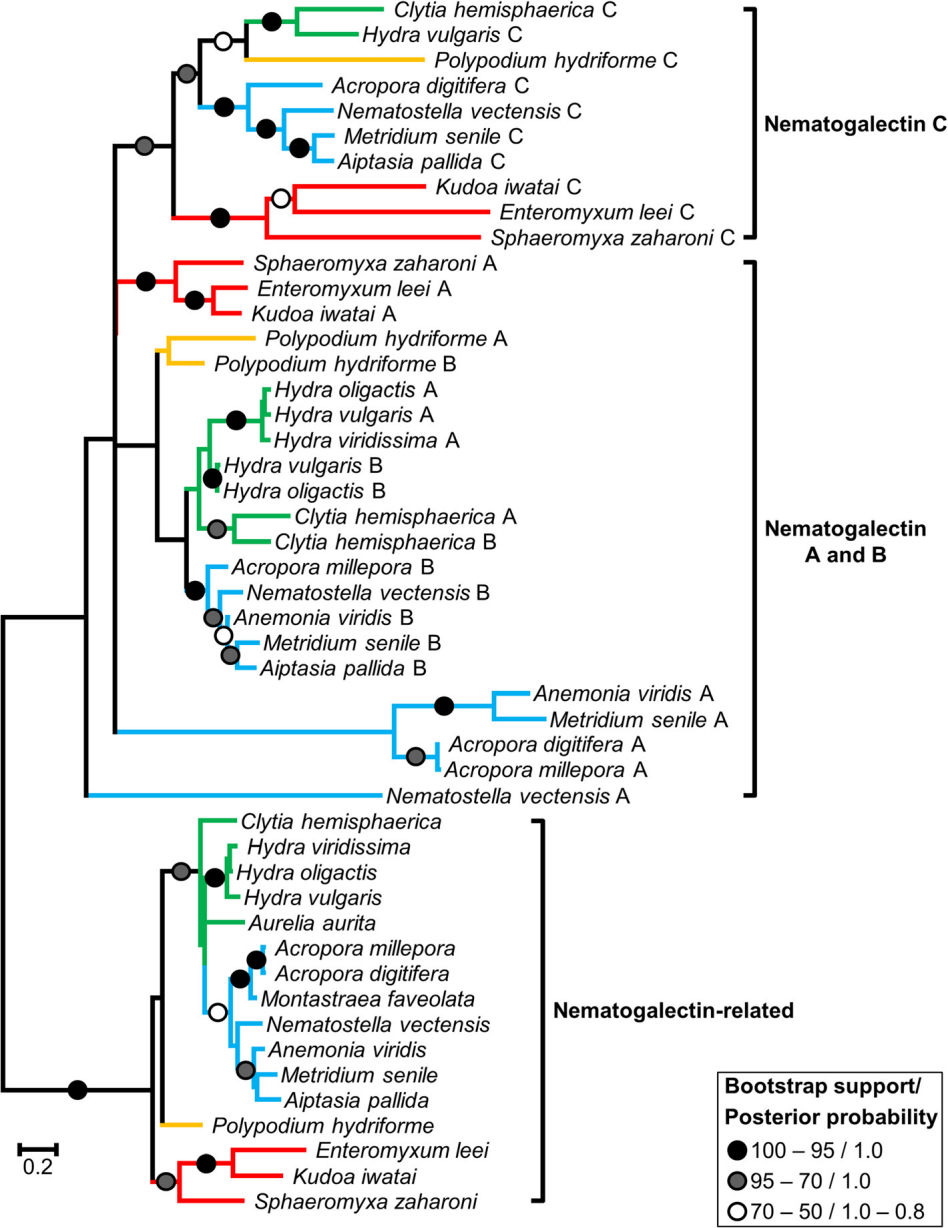
**ML phylogenetic tree reconstructed based on the nematogalectin dataset.** Circles indicate node supports. Black circles: 100 ≥ BP ≥ 95, PP = 1.0. Gray circles: 95 > BP ≥ 70, PP = 1.0. White circles: 70 > BP ≥ 95, 1.0 ≥ PP ≥ 0.8. Nodes with a bootstrap support below 20% were collapsed. Blue, green, orange and red branches represent Anthozoa, Medusozoa, Polypodioza and Myxozoa respectively.

The phylogenetic tree of nematogalectins divides these genes into two clades, nematogalectin-related proteins and other nematogalectins (Figure 4). Many relationships within the nematogalectin clade are poorly supported. However, the nematogalectin C clade is well-supported (BP = 72, PP = 1.0), which suggests a common origin of these sequences. Within the nematogalectin C clade, Myxozoa appear to diverge first which could be the result of a long-branch artefact since they possess the fastest evolving sequences [37]. Other branchings within this clade follow classical taxonomic relationships.

Relationships among nematogalectin A and B are more obscure and the positions of the myxozoan and *Polypodium* sequences cannot be confidently determined. Surprisingly, nematogalectin A and B are not recovered as two distinct clades. Instead, nematogalectin A and B of *Hydra*, *Clytia* and *Polypodium* cluster with each other (BP = 49/79/46, PP = 0.95/1.0/0.6, for *Hydra*, *Clytia* and *Polypodium* respectively), suggesting that these are paralogs in each of these individual lineages (but see Discussion below) (Figure 4). In contrast, all of the representative anthozoan nematogalactin B genes form a well-supported clade (BP = 100, PP = 1). The, anthozoan nematogalectin A genes do not form a monophyletic clade, but this could be due to their fast evolving sequences and the sequence of *Nematostella* in particular, which appears to be extremely divergent (Figure 4).

**Figure 5:**
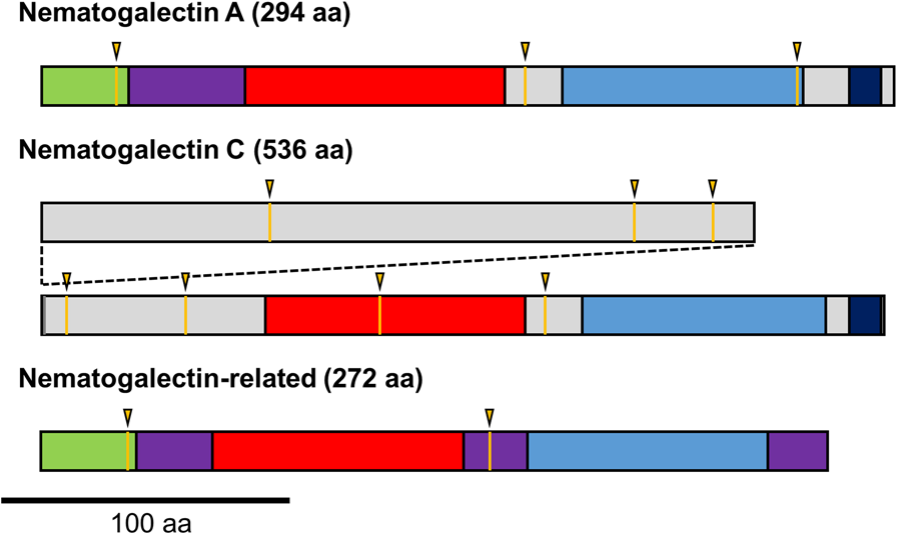
**Schematic drawing of** ***Kudoa*** **nematogalectin and nematogalectin**-**related proteins.** Green: signal peptide; purple: highly conserved areas among nematogalectin of the same type; red: collagen-like domain; blue: gal-lectin-like domain; orange line and triangle: intron-exon boundary.

Among nematogalectin-related genes, the myxozoan sequences (Figure 4, Additional file 1) possess a similar intron/exon structure as in other cnidarians [27] with a first intron after the end of the signal peptide and a second intron between the collagen and the gal-lectin domains (Figure 5). These two introns are also present in nematogalectin A and nematogalectin C (Additional file 1). The nematogalectins A of *Kudoa* and *Sphaeromyxa* possess an additional intron located at the end of the gal-lectin domain, while the nematogalectins C also possess at least five additional introns in their long 5′-region (Additional file 1). No evidence of alternative splicing was found in the CDS of the nematogalectin A, B and the nematogalectin-related genes of *Kudoa* or *Polypodium*. However, although it is possible that alternative splicing occurs in the long 5′-region of nematogalectin C of *Kudoa*, as two different transcripts were found, this could not be confirmed.

## Discussion

Our results indicate that myxozoans possess two cnidarian restricted gene families - minicollagen and nematogalectin - which play a critical role in the nematocyst structure. Like cnidarian proteins, the myxozoan proteins possess signal peptides indicating that they are excreted by post-Golgi vacuoles, in which nematocysts and polar capsules are formed [14,38,39]. The absence of NOWA and Spinalin is not totally unexpected since these two proteins seem to be restricted to *Hydra* as no orthologs were found in the genomes of any other cnidarians [18]. However, we cannot exclude the possibility that unrelated or very divergent proteins might replace NOWA and spinalin in Myxozoa and *Polypodium*.

Our results show that myxozans possess three different minicollagen genes (Figure 2), displaying a higher diversity of minicollagens than previously appreciated. Minicollagens have been divided into three groups based on their cysteine-rich domain (CRD) organization. Specifically, both the N-terminal and C-terminal CRD domains follow the pattern CXXXCXXXCXXXCXXXCC in group 1 minicollagens. Group 2 is characterized by N-terminal CRDs similar to that of group 1 minicollagens but with variable and different C-terminal CRD patterns [21]. Finally, group 3 is characterized by variable CRD patterns both at the N-terminal and C-terminal. Low node support in our phylogenetic analysis precludes us from determining whether this classification reflects orthologous relationships [21] (Figure 2). Following this classification most myxozoans and *Polypodium* genes belong to group 1 minicollagens. However, several exceptions exist. First, *Tetracapsuloides* Ncol-1, which has been considered to be homologous to group 1 minicollagen, would belong to group 3 (see [16]). Additionally, the second and third CRDs of Myxozoan Ncol-2 sequences, and the second CRD of *Polypodium* Ncol-5,6 (which include in Myxozoa and *Polypodium* three and two repeats of the CRD domain in their C-terminal region respectively) are found to possess non canonical CRD domains and thus should be classified as group 2 minicollagens (Additional file 1). Among the additional *Polypodium* sequences, *Polypodium* Ncol-10 possesses a missing non-cysteine residue in its C-terminal CRD and should thus be classified as group 2 minicollagen. Finally, *Polypodium* Ncol-7,8,9 would belong to group 3 minicollagen, Polypodium Ncol-7 is the most derived sequence with non-canonical CRDs at both ends, while *Polypodium* Ncol-8,9 possess a standard CRD domain followed by additional non-canonical CRDs in their N-terminal regions (Additional file 1). These observations confirm that minicollagen structures can be quite variable, among related sequences.

The CRDs of the N- and C- terminal ends of *Hydra* minicollagen NCol-1 (group 1) have been shown to fold into different structures which involve completely different pairs of disulfide bridges among the six cysteine residues [40]. Two non-cysteine residues have been shown to be involved in this conformational change. Precisely, the presence of a valine or isoleucine before the second cysteine and the presence of a proline after the fourth cysteine of the CRD lead to a C-terminal structure in more than 95% of the cases [41]. Interestingly, the presence of only one of these residues has been found to lead to a “bridge state” which can adopt both structures [41].

Surprisingly, myxozoan and *Polypodium* minicollagen canonical CRDs were found to differ from these N- and C- terminal patterns. Specifically, the N-terminal CRDs of Myxozoa NCol-2 and *Polypodium* NCol-6 were found to possess a proline residue after their fourth cysteine (Additional file 1), which would give them the possibility to have a conformation characteristic of a C-terminal end. It is worth noting that these are the only minicollagen sequences which possess such proline mutations in our alignment among canonical N-terminal CRDs. Similarly, the C-terminal CRDs of Myxozoa NCol-1 and *Polypodium* NCol-1-4 do not possess a proline but rather a histidine after their fourth cysteine (Additional file 1), which would suggest that these proteins adopt an N-terminal configuration at both ends. We stress again that these are the only canonical C-terminal CRDs which possess this peculiarity in our alignment. These observations indicate that myxozoan and *Polypodium* minicollagens have novel organizations and might thus form different structures than other cnidarian minicollagens.

The facts that these characteristic structures are shared by *Polypodium* and Myxozoa, and that *Polypodium* and myxozoan minicollagens often belong to the same clades, support the notion that myxozoan minicollagens originated from ancient duplications – prior to the Myxozoa/*Polypodium* divergence - and are not the result of recent specializations of the polar capsule.

The diversity of minicollagens has been linked to nematocyst diversity, under the hypothesis that different minicollagens might be expressed in different types of nematocyst [21]. The life cycle of myxozoans from the class Myxosporea possess two types of spores. Myxospores develop in the vertebrate host (usually a fish) and actinospores develop in an invertebrate (usually a worm). Each type of spore contains polar capsules [1,6]. All three minicollagen genes found in the myxozoan genomes were expressed in the myxospore life cycle stage of the *Kudoa* transcriptome, suggesting that actinospores and myxospores do not possess different minicollagens. This observation agrees with the fact that the polar capsule structure is identical in actinospores and myxospores [6]. The loss of minicollagen diversity in Myxozoa is also in agreement with the hypothesis that the diversity of minicollagen is linked to the nematocyst diversity. By comparison, *Polypodium*, which holds 11 canonical minicollagens, possesses both atrichous isorhizas and four types of holotrichous isorhizas [29]. Similarly *Hydra* possess 21 different types of minicollagens [19] and four different types of nematocysts: desmonemes, atrichous isorhizas, holotrichous isorhizas; and stenoteles [30,31]. This indicates that the myxozoan minicollagen repertoire was dramatically simplified with their parasitic life style.

Similarly, the myxozoan nematogalectin repertoire is also simplified. While cnidarians possess both nematogalectin A and nematogalectin B, myxozoans possess only nematogalectin A. It has been previously shown that nematogalectin genes A and B, are expressed in different parts of the nematocyst tubule in *Hydra* [27]. Specifically, nematogalectin B has been found to be expressed in the proximal tubule, which is also characterized by the presence of numerous barbs or spine ridges, while nematogalectin A is expressed in the distal tubule which is thinner and smoother [27]. Nematocysts whose tubules are entirely spiny (i.e., desmonemes) only express nematogalectin B [27]. This suggests that the polar filament might have a simpler structure than the nematocyst tubule. Interestingly, it is worth noting that the myxozoan polar filament does not possess spines [42], supporting the idea that myxozoans lost the nematogalectin B gene while retaining the nematogalectin A gene.

Our phylogenetic results also show that the nematogalectin A and B sequences cluster as sister groups in *Hydra*, *Polypodium* and *Clytia* (Figure 4), suggesting gene duplications in each of these lineages. However, given their critical role in nematocyst structure (24) and the unparsimonious scenario that each lineage underwent a duplication independently, we suggest that instead, nematogalectin copies are evolving under some level of concerted evolution in medusozoans. This is supported by the observation that in *Hydra*, both nematogalectin copies belong to the same transcript [27]. Such close positioning is known to facilitate concerted evolution in duplicated genes [43]. Conversely, the anthozoan nematogalectin A and B form two distinct clades and it is worth noting that these two genes are located on different contigs in the *Nematostella* assembly [44].

Although no evidence of concerted evolution was found among other nematogalectin genes, we observed that all three genes are present on the same contig in *Sphaeromyxa*. On this contig, the nematogalectin-related gene has a direct orientation and is followed by the nematogallectin A gene which has the same orientation. The nematogalectin C is next but with a reverse orientation. Although our phylogenetic analysis did not recover close relationships amongst these genes in *Sphaeromyxa*, it is possible that some level of concerted evolution might also occur between the nematogalectin related and the nematogalectin A gene in *Sphaeromyxa*.

## Conclusions

Our findings provide compelling evidence in support of the hypothesis that the myxozoan polar capsule is homologous to the cnidarian nematocyst. We here describe nematocyst-specific orthologs from two protein families. However, it is likely that the homology extends to a larger number of proteins.

The origin of the polar capsule, however, is still unclear. The phylogenetic analysis of minicollagen sequences suggests a possible relationship between *Polypodium* and myxozoans and thus a medusozoan origin [12]. However, this sister clade relationship was not found with the nematogalectin sequences, where Myxozoa appear to be an early diverging taxa in all nematogalectin clades. Because myxozoan sequences are fast evolving, it cannot be excluded that this basal positioning is the result of a long-branch artefact. Additional information from the polar capsule genes of divergent medusozoan families and from malacosporean myxozoans should provide further insights into the evolution of the polar capsule.

## Methods

### Myxosporean plasmodia collection

*Kudoa iwatai* plasmodia were isolated from the intracranial adipose tissue of the eye periphery of gilt head sea bream (*Sparus aurata* L.). *Enteromyxum leei* trophozoites were isolated from the intestinal epithelium of infected gilt head sea bream. Finally, large *Sphaeromyxa zaharoni* plasmodia were removed intact from the gall bladder of infected devil firefish *Pterois miles* (Bennett, 1828) (NPA collection permit 2010/37891). All samples were fixed in 100% ethanol or RNA later and kept at −20 C until DNA extraction.

### *Polypodium* collection

Oocytes infected with *Polypodium hydriforme* were collected from mature, female paddlefish (*Polyodon spathula*) at the Paddlefish Research and Processing center in Twin Bridges State Park, Miami, OK. In order to minimize possible contamination from the host, individual stolons of *P. hydriforme* were allowed to emerge from the host oocytes and kept in spring water for 24–48 hours to allow for the paddlefish yolk inside the stolons to be digested. After this period, *Polypodium* specimens were flash-frozen in liquid nitrogen and subsequently kept at −80 C until extraction.

### DNA/RNA isolation and sequencing

Although it is preferable to sequence both the transcriptome and genome for gene identification in non-model organisms, our choice of sequencing was determined by the availability and quality of the material. Unfortunately, we have been unable to obtain good RNA extracts for *E. leei* and *S. zaharoni* and although we have performed genome sequencing in *Polypodium*, sufficient coverage, repeats and large intra and intergenic regions have precluded obtaining a useful assembly.

Before DNA extraction, plasmodia were rinsed in phosphate buffered saline (BPS). DNA was extracted using the Qiagen DNeasy Blood & Tissue Kit following manufacturer instructions. The 18S rRNA gene was amplified and sequenced to confirm the species of the parasite. For *E. leei* the PCR primers used were 18S10 = 5′-TCATTCAATAACATCCACCGAT-3′ and 18S12 = 5′-ATTAGTCATTACCTTGGTTCCGAAA-3′. For *S. zaharoni*, the PCR primers used were 18S1 = 5′-AACCTGGTTGATCCTGCCA-3′ and ERI-B10 = 5′-CTTCCGCAGGTTCACCTACGG-3′. Finally, for *K. iwatai* the primers used were 18S1 and 18S2 = 5′-TGCAGGTTCACCTACAGAA-3′.

RNA extraction of *K. iwatai* plasmodia was performed with the Epicentre kit (MasterPureTM RNA purification kit #MCR85102) following the manufacturer instructions with an extra rinsing with the T&C buffer to remove the RNA later.

For *P. hydriforme*, RNA was extracted from multiple individuals of the elongate stolon developmental stage using TriReagent (Life Technologies, Grand Island, NY) following standard protocols. Extraction was followed by DNAse treatment using the TURBO DNase kit (Ambion). *P. hydriforme* samples were prepared for sequencing using the TruSeq RNA Sample Preparation Kit v2 (Illumina Inc., San Diego, CA). RNA was chemically fragmented, using reagents supplied in the TruSeq kit, resulting in libraries with a median insert size of 155 bp. For contamination filtering for *P. hydriforme*, libraries from uninfected paddlefish eggs were prepared and sequenced at the Genome Sequence Facilty and the University of Kansas Medical School.

The myxozoan samples were then sent for a library construction and sequencing to the Genome Sequencing & Analysis Core Resource of Duke University (Durham, NC). A single lane was used for each of the DNA and RNA samples of *Kudoa*, while two lanes were used both for *Enteromyxum* and *Sphaeromyxa* DNA libraries. *Polypodium* samples were sequenced in single lane at the University of Massachusetts Medical School Molecular Biology Core Facility. Paired-end sequencing of 100 bp reads derived from fragments of average length of 185–280 bp (depending on the library) for the myxozoans and 155 bp for *Polypodium*, was performed on a HiSeq 2000 platform. Reads that failed the chastity and purity quality filtering of the Illumina’s CASAVA pipeline were discarded.

### DNA and RNA assemblies

In order to filter out any host contaminants from the *Polypodium* transcriptome, a transcriptome of uninfected oocytes from the host (paddlefish) was sequenced and assembled (see below). Reads from the *Polypodium* transcriptome were mapped to the resulting paddlefish assembly using the “—very sensitive” settings of Bowtie2 [45]. Reads which did not map to the oocyte sequences (i.e., reads which were not paddlefish contaminations) were then used to build the *Polypodium* transcriptome assembly. Filtered reads from *P. hydriforme* were examined in FastQC v.0.8.0 by Babraham Bioinformatics (2010) (http://www.bioinformatics.babraham.ac.uk/projects/fastqc/) for overall quality score distributions and to assess quality cutoffs. Reads were quality trimmed with a script created by M. Shcheglovitova (https://github.com/bastodian/shed/blob/master/Python/q-trim.py) using default values of Phred score cutoff of 21, 5 bp of contiguous low quality sequence and at least 30 bp left of a sequence in order for the read to be retained. The *P. hydriforme* and paddlefish trimmed reads were assembled using Trinity r20121005 [36]. Each run was implemented using the colony. bash wrapper script for Trinity, written by Paul Calnon at the University of Kansas Advanced Computing Facility’s Community Cluster. The script was run with the following settings for both assemblies: (‐‐SS_lib_type RF ‐‐CPU 10 ‐‐min_contig_length 200 ‐‐bflyJavaVM64bit ‐‐bflyHeapSpace 20G ‐‐bflyMinHeapSpace 20G ‐‐bflyHeapNursery 20G ‐‐bflyJavaGCParallel ‐‐bflyJavaGCThreads 16 ‐‐repeat 5 ‐‐bflyJavaCmdLifespan_min 5 ‐‐bflyJavaCmdLifespan_max 1800 ‐‐bfly_opts "-V 10 ‐‐stderr") on the Bioinformatics Cluster at the Information and Telecommunications Cluster at the University of Kansas. After assembly, contigs with low support were filtered from the *Polypodium* transcriptome assembly using the RSEM pipeline (v1.2.13). The reads used for this assembly were mapped to the assembly using Bowtie and then the RSEM algorithm was used to estimate expression values, using the align_and_estimate_abundance.pl script packaged with Trinity r20140413. Contigs were filtered based on the estimated expression values using, also packaged with Trinity, using the follow settings: ‐‐fpkm_cutoff = 0.01 ‐‐isopct_cutoff = 1.00. Out of the contigs that passed this cutoff, only those longer than 300 bp were kept for future analysis. This Transcriptome Shotgun Assembly project has been deposited at DDBJ/EMBL/GenBank under the accession GBGH00000000. The version described in this paper is the first version, GBGH01000000.

The myxozoan DNA reads were assembled with the ABySS *de novo* assembler [46] using a *k*-mer length of 64, a minimum number of 10 pairs required for building contigs, and all other parameters remaining with their default values. Host contaminations could not be filtered from the myxozoan assemblies. However, because we only focus on the characterization of cnidarian specific genes fish contaminations are not a concern in this wok.

The *Kudoa* RNA reads were assembled using the Trinity *de novo* assembler version r20131110 [36], using the three following flags: ‐‐min_contig_length 100 (setting the shorter contigs length to 100 bp has been found to improve the quality of the assembly) ‐‐min_kmer_cov 2 (this option has been shown to reduce the number contaminant and low quality contigs) ‐‐jaccard_clip (this option reduces the level of fused transcripts, and is adapted for compact genomes such as parasite genomes) [47].

### Blast searches of cnidarian nematocyst genes against RNA assemblies

Searches were conducted on The National Center for Biotechnology Information (NCBI) protein database with the queries ‘minicollagen Cnidaria’ , ‘nematogalectin’ , ‘spinalin’ and ‘NOWA Cnidaria’. All proteins were downloaded and redundant sequences were removed. Thirty tree minicollagen sequences, 14 nematogalectin sequences, one NOWA sequence and one spinalin sequence were then used as queries to conduct tBlastn searches with default settings against the RNA assembly of *K. iwatai*, and *P. hydriforme*. Positive hits were only obtained for minicollagen and nematocyst proteins. All contigs that gave a positive hit with any of the query sequences were translated into protein sequences. A reciprocal Blastp search was then conducted against the whole protein database of NCBI to confirm the hits did not belong to a different protein family.

Minicollagen sequences identified in myxozoans and cnidarians were then used as query to conduct tBlastn searches against myxozoan EST data available in NCBI. Similarly, nematogalectins sequences identified from myxozans and cnidarians were then used as queries to conduct tBlastn searches against cnidarian EST and protein data available in NCBI. Blast searches were also conducted against the ZoophyteBase to identify *Acropora digitifera* sequences [48]. All minicollagen and nematogalectin contigs, longer than 100 bp that gave a positive hit with any of the query sequences were translated into protein sequences. Identical protein sequences were removed by hand, and reciprocal Blast searches were conducted as indicated above.

### Blast searches of cnidarian nematocyst genes against DNA assemblies

Protein sequences extracted from NCBI and RNA assemblies, as indicated above, were used as queries to conduct tBlastn searches with default settings against the DNA assemblies of *K. iwatai*, *S. zaharoni* and *E. leei*. For *K. iwatai* DNA contigs that gave a positive hit were aligned using MAFFT (version 7) [49] with default settings against the corresponding RNA sequences. This allows us to identify the intron-exon boundaries of most genes, except for the 5′ region of the nematogalectin C. For the other two myxozoan species and for the *Kudoa* nematogalectin C we used the gene prediction webserver Augustus (http://bioinf.uni-greifswald.de/augustus/submission.php) [35] to detect the intron exon structure of minicollagen and nematogalectin contigs that gave a positive hit. The species *Bombus terrestris*, *Apis melifera* and *Acyrthosiphon pisum* were selected as model, since these organisms were found to give the best gene prediction results. When Augustus failed to identify a complete protein coding gene, intron-exon boundaries were identified manually using information from *Kudoa* sequences. Signals peptides were predicted using the SignalP 4.1 Server (http://www.cbs.dtu.dk/services/SignalP/) [50] with the options D-cutoff values set to “sensitive” and Method set to “input sequence do not include TM regions”. Signals peptides were identified in all nematogalectin sequences except the nematogalectin-C and the nematogalectin A of *Sphaeromyxa*. Myxozoa sequences have been submitted to the EMBL-EBI European Nucleotide Archive under accession numbers LK936446- LK936461.

### Phylogenetic reconstructions

Minicollagen proteins are short and can possess several alternative transcripts [18], consequently the exact number of minicollagen genes is not always easy to determine from transcriptome data. We thus chose to include in our phylogenetic analysis only sequences that diverged by more than five amino acids and follow the canonical minicollagen structure: a signal peptide, a pro-peptide, a first cysteine-rich-domain (CRD), a first proline repeats, a collagen-like domain formed of GXY repeats, a second proline repeats, a second CRD and often a basic tail (Figure 3) [20,51]. For example, among the 21 described minicollagen of *Hydra* [19], only 16 were found to follow the canonical stucture.

The canonical nematogalectin structure includes –a signal peptide, a conserved region, a collagen-like domain formed of GXY repeats, a sugar-binding gal-lectin domain and often a basic tail rich in lysine (Figure 5) [27]. We included in our analysis all sequences following these pattern as well as sequences which did not possess a conserved domain in their N-terminal region.

The protein sequences of 56 minicollagens and 49 nematogalectin were aligned with MAFFT (version 7) [49] under the Einsi algorithm. Following Holland et al. [16], all amino-acid positions were kept in the minicollagen analysis (Additional file 3). In the nematogalectin analysis, we used the Guidance web-server to detect unreliable residues in sequences [52]. Residues with a treshold below 0.250 were masked (i.e., replaced by X). Additionally, the long fast evolving region present only in nematogalectin C was excluded from the analysis (Additional files 2 and 4). The best models of protein evolution were identified to be Blossum62 + I + G + F and the WAG + I + G for the minicollagen and nematogalectin datasets respectively using ProtTest (version 2.4) [53]. Maximum likelihood ML reconstructions were performed with PhyML (version 3.1) [54] under the model identified by ProtTest. The ML searches were conducted using 50 random starting trees, “best of NNIs and SPRs” branch swapping option, and branch support was determined after 100 bootstrap replicates. Bayesian analyses were conducted with MrBayes (version 3.2) [55] also under the model identified by ProtTest. For both data sets, two runs with eight chains each were conducted, under the ProtTest model, with default parameter for 50,000,000 and 40,000,000 generations for the minicollagen and nematogalectin datasets respectively. The chains were sampled every 100 generations. The initial 25% of the total generation were discarded as burnin, after having verified that log likelihood values of both chains converged before the burnin threshold. At the end of the run, the average standard deviation of split frequencies was below 0.001 for the minicollagen and nematogalectin datasets respectively. We also verified that the potential scale reduction factor (PSRF) were close to 1.0 for each parameter.

## Additional files

Additional file 1:
**Color-coded alignments of minicollagen and nematogalectin proteins from** ***Polypodium*** **and Myxosporea.**
Additional file 2:
**Nematogalectin protein alignment.** Complete Protein sequence alignment, in Nexus format, without any masking. Additional file 3:
**Minicollagen protein alignment.** Protein sequence alignment, in Nexus format, used to reconstruct the phylogenetic tree present in Figure 2. Additional file 4:
**Masked nematogalectin protein alignment.** Masked protein sequence alignment, in Nexus format, used to reconstruct the phylogenetic tree present in Figure 4.
