# Supplementary material for: Diversity and evolution of myxozoan minicollagens and nematogalectins
Source: BMC Evol Biol. 2014 Sep 29;14:205. doi: 10.1186/s12862-014-0205-0 (PMC4195985; doi:10.1186/s12862-014-0205-0)
Supplement: Additional file 1: — Color-coded alignments of minicollagen and nematogalectin proteins from Polypodium and Myxosporea. [file 12862_2014_205_MOESM1_ESM.docx]

**Diversity and evolution of myxozoan minicollagens and nematogalectins**

Erez Shpirer, E. Sally Chang, Arik Diamant, Nimrod Rubinstein, Paulyn Cartwright, Dorothée Huchon.

Additional file 1

Polypodium_hydriforme_Ncol-7 ----MMSYGWVLIGLVAVTSAMSLDKRSAEP-----**C**DGAG**C**GG**C**GD**C**-----YGAGGYGAPSYGVGGYGG**C**PPS**C**ASG---------------------

Polypodium_hydriforme_Ncol-8 --MSLIFAFSLAVVAVSGVWSAALEKREAEP-----**C**GYG**C**PSY**C**APS**C**SSS**CC**----------------------------------------------

Polypodium_hydriforme Ncol-9 ---MCTFVVLSVLVLVSEMSAMTLDKRSADA-----**C**GYG**C**SPS**C**APS**C**NPQ**CC**SYMINPVPPPPPPMAPT**C**MYPSS**C**YAPAPPA**C**MASPM**C**--------

BPE00000549_BuddenbroCkia_plum ---MKLILGILLLTYLIDVYGEKSLFRRQVNT----**C**SPG**C**PTS**C**YPE**C**TPT**CC**----------------------------------------------

Enteromyxum_leei_Ncol-3 -MFKEVSGLVLFLSTIALVRADK**G**DKVFKRSPQYDS**C**GPA**C**PPT**C**APS**C**SVQ**CC**----------------------------------------------

Kudoa_iwatai_Ncol-3 --MIRGVFGLLLSTVALSFAATEA**E**KVYKRSPQVNV**C**GP**VC**PPI**C**APA**C**TVQ**CC**----------------------------------------------

Polypodium_hydriforme_Ncol-11 ----MAMFLPLLLLVWVGAEAKSLHEMLRREANP--**C**GSA**C**PSY**C**APS**C**LTS**CC**----------------------------------------------

Sphaeromyxa_zaharoni_Ncol-3 ----MNHLSLLLVSAIVVVHSKNID**G**VFKRSSYP--**C**GYP**C**PIS**C**APA**C**LPA**CC**--------------------------------------AAPAPAPV

Enteromyxum_leei_Ncol-1 -----MLTLSLLFCFISYTFASLPKSV**E**KRSPQL--**C**DAA**C**PAY**C**APA**C**TPI**CC**----------------------------------------------

Kudoa_iwatai_Ncol-1 ---MVMFTLPILVCLFSYTLASLPRSS**E**KRSPQAV-**C**DYG**C**PAV**C**APA**C**LPV**CC**----------------------------------------------

Polypodium_hydriforme_Ncol-2 ----MIHRSVVLLALVAVASCGLPRNIEKRSPQS--**C**DLG**C**QAV**C**APT**C**LPI**CC**----------------------------------------------

Polypodium_hydriforme_Ncol-4 ----MASYLVCVIALIGSISAGIPRENEKRSPQF--**C**DAG**C**PSY**C**APA**C**QPI**CC**----------------------------------------------

Polypodium_hydriforme_Ncol-1 ---MLTRLSVPLMLLLGVAFAGIPRELEKRSPQY--**C**DSG**C**PSY**C**APS**C**QPI**CC**----------------------------------------------

Polypodium_hydriforme_Ncol-3 ----MEYSLVLLSALLGVSFAGLPRTLDKRSPDF--**C**GAP**C**PSF**C**APS**C**LPV**CC**----------------------------------------------

Sphaeromyxa_zaharoni_Ncol-1 -----MLSSLIASILIPFTISGLPRVK**E**KRQVYPY-**C**PAP**C**PAT**C**APA**C**LPV**CC**----------------------------------------------

CBJ19442_TetraCapsuloides_bryo ----MLLKLLLSFTTYNIINCGIPQSLEKRSLDV--**C**SY-**C**PVQ**C**QPT**C**SPV**CC**----------------------------------------------

Polypodium_hydriforme_Ncol-10 ---MNRVILLALFGTLCWAMVSSMPN----------**C**GSG**C**SSS**C**APT**C**SYG**CC**----------------------------------------------

Polypodium_hydriforme_Ncol-5 ----MILIISIGLLYLAGINANALNVRPERQLS---**C**SGG**C**PGY**C**APQ**C**MPS**CC**-------VPPPPPPPVL**C**PQT**C**AVS**C**APP**CP**VV**CC**-----------

Polypodium_hydriforme_Ncol-6 MSLHGLLTWIIVSLALTHLAAAAQPKNGSPVSRQLI**C**PES**C**TQM**C**TPA**CP**QL**CC**----------------------------------------------

Enteromyxum_leei_Ncol-2 --MWNTLLLLILSSHLYVTEPILS**K**YNEKKQRIAV-**C**SPA**C**ESQ**C**IPT**CP**AV**CC**----------------------------------------------

Kudoa_iwatai_Ncol-2 --MGVGIDVTLLLVLIIYAHPSYT**K**NPTKKQYINQ-**C**PP**IC**ATN**C**VPA**CP**AL**CC**----------------------------------------------

Sphaeromyxa_zaharoni_Ncol-2 ----MFVTALVGINFLLISFSVPLK**E**VLKRHIGIS-**C**PPL**C**QSY**C**YSY**CP**PT**CC**----------------------------------------------

BPE00000585_BuddenbroCkia_plum -MINNELKIMRILTIISTLSCLSTHYYVQRENS---**C**QNX**C**PLR**C**YPS**C**LPN**CC**----------------------------------------------

110 120 130 140 150 160 170 180 190 200

....|....|....|....|....|....|....|....|....|....|....|....|....|....|....|....|....|....|....|....|

Polypodium_hydriforme_Ncol-7 ---------------------------------PGMIAAGPQGSPGAMGFPGPMGPPGAPGFMGPPGPMGPPGVPGFPGVPGAPGAS-------------

Polypodium_hydriforme_Ncol-8 -------GYAAYPAPAPA**C**GYASAPA**C**APAAAAAPMMIPGPPGAPGMMGSPGFMGPAGAPGMMGAPGPMGPPGSPGMPGAPGAPGAS-------------

Polypodium_hydriforme Ncol-9 -------------------------------ASLPASIPGPPGPPGCMGPMGSPGCAGLMGAPGMPGAPGMPGAPGVPGAPGVPGAS-------------

BPE00000549_BuddenbroCkia_plum ---------------APQQQVYYPPPPPPPPSPPIPALPGPPGPPGRPGAMGPMGPPGMQGPMGPPGQQGSPGSPGIPGSPA------------------

Enteromyxum_leei_Ncol-3 -APPPPPPPPPPPPPPPPPVYYPPPPPPSPPPPPLPALPGPPGPPGKPGSAGLMGPPGVAGPPGPPGPPGVSGTPGAPGAPA------------------

Kudoa_iwatai_Ncol-3 ------TAPPPPPPPIYIPPPPPPPPPPPPPPPPLPALPGPPGPPGKPGPAGLMGPPGPQGPPGPPGPPGISGTPGAPGAPA------------------

Polypodium_hydriforme_Ncol-11 -----------------------------APAGPIPALPGPPGPPGRPGPPGPMGMPGMPGPPGPPGAPGASGSPGTPGAPA------------------

Sphaeromyxa_zaharoni_Ncol-3 YVAPAPAPVYVPPPAPPVYVPPPPPPPPLPPLPPLPALPGPPGMPGKPGPSGLMGPPGPPGAPGAPGAAGQPGVPGQPGAPA------------------

Enteromyxum_leei_Ncol-1 ---------------------------APAAPALPPPPPGPMGQSGQPGQPGPIGPPGPPGPPGPPGPSGSSGSPGYPGAAA**G**VP---------------

Kudoa_iwatai_Ncol-1 --------------------------VAAAAPALPPPPPGPQGSPGPAGQPGPQGPPGPPGPPGPPGLPGGAGSPGQPGAAAGPPGPNGP**AG**PMGPRGNM

Polypodium_hydriforme_Ncol-2 ------------------------------LPPPPPPPPGPPGSPGPVGLSGPSGPPGPPGAPGSPGLPGLPGPVGLPGAAAGSPGVNGPQGPQGGNGPQ

Polypodium_hydriforme_Ncol-4 ----------------------------IPVPPPPPPPPGPPGSPGGPGLPGPFGPPGPPGPPGFPGQPGFPGPVGLPGAAAGLPGVNGPQGPMGPVGPP

Polypodium_hydriforme_Ncol-1 ----------------------------IPAPPPPPPPPGPPGSPGNVGLPGPFGPPGPPGIPGIPGFPGQPGSPGVPGAPAGIPGVNGPQGPQGSAGPP

Polypodium_hydriforme_Ncol-3 ----------------------------IPAPPPPPPPPGPPGSPGGVGLPGPFGPPGLPGPPGPPGPPGYPGPLGLPGAPAGIPGVNGPQGPQGLNGPP

Sphaeromyxa_zaharoni_Ncol-1 ----------YSMAAVPAVAAIPAVAAVPALPPLPPPPPGPMGQPGPIGPPGPPGPPGLPGVQGMPGPLGSAGSPGYPGAAAGIPGPNGA**PG**PLGAPGFM

CBJ19442_TetraCapsuloides_bryo -------------------------------VSPPPPPPGPPGIPGPYGQMGPPGPPGPPGFSGSPGLPGFPGPQGLPGAPAGPPGINGSPGMIGPRGQP

Polypodium_hydriforme_Ncol-10 ---------------------------------PVQAPPGAPGSPGPMGLMGPPGPVGPGGPPGNPGPAGPPGNPGTPGLP-GNPGLMGPPGPPGPPGLP

Polypodium_hydriforme_Ncol-5 ----------------------------LPAPPPPPPPPGPPGAPGPAGLPGPYGPPGFPGPIGPMGPPGPPGPPGQPGIAL------------------

Polypodium_hydriforme_Ncol-6 ----------------------------QLQALPPPPPPGPPGANGPPGLPGPPGPSGPRGLPGPPGEMGQPGLPGPPGVQA------------------

Enteromyxum_leei_Ncol-2 --------------------------------NLPAPPPGPPGIPGQVGIPGQPGPNGPPGPVGPQGPPGPPGSPGMPA---------------------

Kudoa_iwatai_Ncol-2 ---------------------------------LSPPPPGPVGSPGPPGLPGPQGPNGPPGPPGPPGPPGPAGSPGEPA---------------------

Sphaeromyxa_zaharoni_Ncol-2 -------------------------APL---PPLPPPPPGPMGQPGPPGLAGPQGPPGPPGPPGRPGIPGFRGSPGLAGIPA------------------

BPE00000585_BuddenbroCkia_plum ------------------------------SPIPPPPPPGPPGIPGPQGLTGPVGLPGLMGPPGQPGLAGQPGLPGNPGQQGPP----------------

210 220 230 240 250 260 270 280 290 300

....|....|....|....|....|....|....|....|....|....|....|....|....|....|....|....|....|....|....|....|

Polypodium_hydriforme_Ncol-7 -----------------------------**C**PP**IC**ITH**C**MRI**CP**LS**CC**--------------------------------------TASPLPPPPPPM**C**MP

Polypodium_hydriforme_Ncol-8 -----------------------------**C**PP**IC**VTH**C**MRI**CP**LP**CC**--------------------------------------APPPPPPPPQMA**C**AM

Polypodium_hydriforme Ncol-9 -----------------------------**C**PP**IC**IQH**C**MRI**CP**MS**CC**----------------------------------------APPPPPPPPV**C**MP

BPE00000549_BuddenbroCkia_plum ------------------------PPPKP**C**QPS**C**ATN**C**IMA**CP**QY**CC**-----------------------------------------------------

Enteromyxum_leei_Ncol-3 ------------------------PPPVQ**C**PSS**C**ITQ**C**TQS**CP**MY**CC**-----------------------------------------------------

Kudoa_iwatai_Ncol-3 ------------------------PPPAP**C**PVF**C**QTR**C**VDS**CP**LY**CC**-----------------------------------------------------

Polypodium_hydriforme_Ncol-11 ------------------------PPPAP**C**PSS**C**QSQ**C**VSS**CP**MY**CC**-----------------------------------------------------

Sphaeromyxa_zaharoni_Ncol-3 ------------------------PPPAP**C**PP**IC**ATQ**C**VMD**CP**LY**CC**-----------------------------------------------------

Enteromyxum_leei_Ncol-1 GRPGLPGPPGPPGAPGAPGAPAPPPPPPP**C**PLM**C**TRK**C**VET**C**HPQ**CC**-----------------------------------------------------

Kudoa_iwatai_Ncol-1 GQPGLPGPPGPPGPPGLPGAPAPPPPPPP**C**PY**VC**TKT**C**TTS**C**HPT**CC**-----------------------------------------------------

Polypodium_hydriforme_Ncol-2 GPPGLPGPPGPPGRPGLPGAPAPPPPPPP**C**PV**VC**TVQ**C**TRT**C**HPT**CC**-----------------------------------------------------

Polypodium_hydriforme_Ncol-4 GRPGLPGPPGQPGRPGMPGAPAPPPPPPP**C**PV**VC**TMT**C**TKT**C**HPT**CC**-----------------------------------------------------

Polypodium_hydriforme_Ncol-1 GGPGLPGPPGPPGRPGSPGAPAPPPPPPP**C**PV**VC**TMQ**C**TKT**C**HPT**CC**-----------------------------------------------------

Polypodium_hydriforme_Ncol-3 GPPGRPGPPGPPGRPGFPGQPAPPPPPPP**C**PV**VC**TLT**C**TPT**C**HPT**CC**-----------------------------------------------------

Sphaeromyxa_zaharoni_Ncol-1 GPPGPPGPPGPPGPSGLPGAPAAPPPPPP**C**PY**VC**TTT**C**LPT**C**HPT**CC**-----------------------------------------------------

CBJ19442_TetraCapsuloides_bryo GQPGLPGSPGPNGMPGASGLPAPPPPPPP**C**PY**VC**TTRPA**C**L**C**HPT**CC**-----------------------------------------------------

Polypodium_hydriforme_Ncol-10 ------------------------APPAP**C**PQT**C**MTS**C**MT-**CP**NY**CC**-----------------------------------------------------

Polypodium_hydriforme_Ncol-5 ------------------------PPPPV**C**TRT**C**YTY**C**YET**CP**QY**CC**QQ----PAT-----------------------------------PPPPPV**C**PP

Polypodium_hydriforme_Ncol-6 ------------------------PPPQV**C**TFS**C**YQS**C**VPT**CP**SY**CC**TRPVAQVYS-------------------------------PPPPPPPPPP**C**PP

Enteromyxum_leei_Ncol-2 ---------------------PPSPPVKM**C**TME**C**LTT**C**APS**CP**TY**CC**PQEVVSTTPPPPV---**C**PP**IC**TVTT**C**ISS-**CP**SD**CC**---QPPAPTPSTSN**C**PA

Kudoa_iwatai_Ncol-2 ---------------------PQAPPPQI**C**PLS**C**YTE**C**VET**CP**QY**CC**VGPMPSPPPPPPPQIV**C**PPT**C**TVDV**C**AID-**CP**TE**CC**--VQPPPPPPTSLV**C**PP

Sphaeromyxa_zaharoni_Ncol-2 ------------------------PPPQV**C**PVS**C**YTV**C**APT**CP**TY**CC**AAPPPPPPPPV-----**C**PA**IC**ETT-**C**API-**CP**PV**CC**------LPPTPPVA**C**PP

BPE00000585_BuddenbroCkia_plum --------------------------LEI**C**KIE**C**YQT**C**SDS**CP**KY**CC**SNSQTQPT--------**C**PDF**C**SQQ-**C**VPGV**CP**NS**CC**TNVPAELAQVQTQP**C**PE

**Fig. S1.** Myxozoan and *Polypodium* minicollagens. The sequence alignment was performed using Mafft and corrected manually. Minicollagen proteins contain a signal peptide (green-shaded areas) at their N termini. A collagen domain made of repeats of tripeptide GlyXY (red-shaded areas) is flanked on both sides by proline repeats (cyan-shaded areas) and cysteine-rich domains (yellow-shaded areas). Cysteine residues of the CRDs are indicated in red. The proline and valine/isoleucine residues that are responsible of CRD conformation change are indicated in dark bold. Residues that span two exons (either a single residue spanning the exon-exon junction or two residues on each terminus of an exon), when intron positions are known, are indicated in gray. (Continued on the next page.)

310 320 330 340

....|....|....|....|....|....|....|....|....|..

Polypodium_hydriforme_Ncol-7 AP**C**SPPSY**CC**-----------------G-----------------

Polypodium_hydriforme_Ncol-8 PS**C**MPPPPPPM**C**MPQP**C**SPPSP**CC**---G-----------------

Polypodium_hydriforme Ncol-9 AP**C**APPPP**C**MAPP**C**AMQTP**CC**------G-----------------

BPE00000549_BuddenbroCkia_plum ---------------------------PVV---------------

Enteromyxum_leei_Ncol-3 ---------------------------PARRR-------------

Kudoa_iwatai_Ncol-3 ---------------------------PARR--------------

Polypodium_hydriforme_Ncol-11 ---------------------------PARK--------------

Sphaeromyxa_zaharoni_Ncol-3 ---------------------------PTKK--------------

Enteromyxum_leei_Ncol-1 ----------------------------FKH--------------

Kudoa_iwatai_Ncol-1 ----------------------------AKH--------------

Polypodium_hydriforme_Ncol-2 ----------------------------AKH--------------

Polypodium_hydriforme_Ncol-4 ----------------------------AKH--------------

Polypodium_hydriforme_Ncol-1 ----------------------------YKH--------------

Polypodium_hydriforme_Ncol-3 ----------------------------AKK--------------

Sphaeromyxa_zaharoni_Ncol-1 -----------------------------KH--------------

CBJ19442_TetraCapsuloides_bryo ----------------------------AQK--------------

Polypodium_hydriforme_Ncol-10 -----------------------------SK--------------

Polypodium_hydriforme_Ncol-5 **VC**SYS**C**AV--**CP**VS**CC**------------PAKHRKSTLDQQFKNVQDA

Polypodium_hydriforme_Ncol-6 **VC**FNT**C**VV--**CP**SA**CC**-----------MPGRKRSDFVKHHQADP---

Enteromyxum_leei_Ncol-2 **IC**QAT**C**AI--**CP**SS**CC**-------------KKRKRHHILSSQAQYID

Kudoa_iwatai_Ncol-2 **IC**QVS**C**AV--**CP**TE**CC**-------------TKHRRHHILSTKEKSMD

Sphaeromyxa_zaharoni_Ncol-2 **VC**STT**C**AV--**CP**PI**CC**------------AKHKRQNILSKENIQQEN

BPE00000585_BuddenbroCkia_plum **IC**QTQ**C**IKPL**C**STS**CC**----------SPYFKRTLNDDHDXENNXFI

**Fig. S1 continued.** Myxozoan and *Polypodium* minicollagens. The sequence alignment was performed using Mafft and corrected manually. Minicollagen proteins contain a signal peptide (green-shaded areas) at their N termini. A collagen domain made of repeats of tripeptide GlyXY (red-shaded areas) is flanked on both sides by proline repeats (cyan-shaded areas) and cysteine-rich domains (yellow-shaded areas). Cysteine residues of the CRDs are indicated in red. The proline and valine/isoleucine residues that are responsible of CRD conformation change are indicated in dark bold. Residues that span two exons (either a single residue spanning the exon-exon junction or two residues on each terminus of an exon), when intron positions are known, are indicated in gray.

10 20 30 40 50 60 70 80 90 100

....|....|....|....|....|....|....|....|....|....|....|....|....|....|....|....|....|....|....|....|

Polypodium hydriforme A M------------WWPTLSLLLFCL-LDNHESEGQRMMPEWPHVGDRVSQSFLDQLMVSQLLEQNLTLGFFLKGLNGPPGPPGPTGPAGDPGPPGMPGAP

Polypodium hydriforme B M----------KSLGRILTVFSFCL----ALTVCQRMDGLWPRPGEPVTQSFLDQLMVSQLLEANLTIGFFMKGMPGPPGPPGQPGPPGDPGQDGMPGAP

Enteromyxum leei A M-----HKYNAEFQWNFFKFFLISYI**TI**VSSENFHPQL-NLPQIGDVVTEEMINQLMISNLIAQNMTVGFFLRGLNGPPGPPGPPGEAGQPGEPGSQGPP

Kudoa iwatai A MADQIKNAINLCSLFSTYTYYLIIL**GV**VMSQR--PPQL-NLPQLGDVVDQNLIDQIMISNLVSQNLTMGFFLRGLNGPPGPPGPPGEPGVPGEPGLPGAP

Sphaeromyxa zaharoni A M-----ILINIKPY-----IIGIFFILEIK**HN**QCQRQI-VLPQVGEPITQQMIDQLMISQLLSQNLTMGFFLRGLNGPPGEPGMPGAPGDPGPPGFPGAP

110 120 130 140 150 160 170 180 190 200

....|....|....|....|....|....|....|....|....|....|....|....|....|....|....|....|....|....|....|....|

Polypodium hydriforme A GLPGHVGEDGAPGPIGLQGPPGMAGPPGPPGSKGDTGMSGIPGEPGLPGAPGLPGLPGPMGPPGSDPLMPNFTVICEGEKGWLQCKQYELVKVTRAFWGR

Polypodium hydriforme B GLPGSVGEDGAPGPLGPPGSTGRPGPPGPPGGKGDPGLPGPMGIPGLAGAPGLPGSPGEMGPPGGEVFMPNYTVVCEGEKAWLQCKQYELVKVSRAFWGR

Enteromyxum leei A GLQGQVGEDGAPGPKGPSGEIGAPGAPGLTGPKGDPGEQGIPGAQGPKGDPGDIGPPGMPGSVGSDLIS**PN**YTVICEGEKGWIQCKQYEVVNIIKVFWGR

Kudoa iwatai A GLQGQVGEDGAPGPPGPRGEMGPPGAPGLTGAKGDPGEQGIPGAQGIPGEPGPMGPPGLPGSSTGDLIT**PN**YTVICEGEKGWIQCKQYEVVNVIKVFWGR

Sphaeromyxa zaharoni A GLPGSVGEDGAPGPAGPTGNTGAPGAPGLRGPQGEPGEQGSPGPPGPPGHPGPVGPPGEPGSPAPEIFM**PN**YTVICEGEKAWIQCKQYEVVTINKVYWGR

210 220 230 240 250 260 270 280 290

....|....|....|....|....|....|....|....|....|....|....|....|....|....|....|....|....|....|....|....

Polypodium hydriforme A DDYSTCPNAPAGLTTERLCETGAENTLAKVNNQCKNSQACEVVASNIFFDDNSCGNVFKYLKLWYECIADEANAVDVLRDGNRKKRRQATKDKRNLRDE

Polypodium hydriforme B DDFSTCSDVPAGLVADRLCETSPENTLAKIDDQCKNKQACEIIATNIFFDDNSCGNVFKYLKVWFECLPDEINTTEVHDGSKKKKRRNVRK-----IEA

Enteromyxum leei A DDFSTCEKAPAGLTTERLCETNSDDAFTKINDQCKNTQACEVVATNLFFNDNTCGNVYKYLKLW-----------------------------------

Kudoa iwatai A DDFTTCEKSPAGLTTDRLCETNTDDALAKINDQCKNTQACEVVATNLFFNDNSCGNVYKYLKL**W**YDCVPDEVNAVDVLRDEARKRRRSVKAKRHTVV--

Sphaeromyxa zaharoni A DDYTTCDKVPAGLTKDRLCDANEEEAYEKVVDQCRNKQACEVVATNIFFNDNSCGNVYKFLKI**W**YDCMPDDLNSIDVPKDGEKRRKRWI------IVEN

**Fig. S2.** Myxozoan and *Polypodium* nematogalectins A and B. The sequence alignment was performed using Mafft and corrected manually. Nematogalectin A and B proteins contain a signal peptide (green-shaded areas) at the N terminus. A collagen domain made of repeats of tripeptide GlyXY (red-shaded areas) in the center and a sugar-binding galectin domain (cyan-shaded areas) at the C-terminus. Residues that span two exons (either a single residue spanning the exon-exon junction or two residues on each terminus of an exon), when intron positions are known, are indicated in gray.

10 20 30 40 50 60 70 80 90 100

....|....|....|....|....|....|....|....|....|....|....|....|....|....|....|....|....|....|....|....|

Polypodium_hydriforme MTLPAMS--FRRMVPKWAWLVHSILLIVIFAQPAVSVPQFQVPPLLNQLLRDQNVTLGFILKGLQGPPGKDGLPGMPGQPGLMGPQGMPGDPGGPGAPGL

Sphaeromyxa zaharoni MNIIA-----PRKIFKFKFFV-CCLIIFYVNF**KV**VSNQSLPIPPLLDQLLRDQNVTLGFILKGLQGPPGMDGSPGYPGPPGLPGPIGFTGEMGPMGPPGS

Enteromyxum leei MVFWYYSETSSKIIWKLCI----IIYLSFLY**RI**NAQN--SSIPPLLNQLLKDQKVTLGFILKGLQGPPGMDGMQGPPGMIGPGGPPGMTGEMGPMGPPGM

Kudoa iwatai MIFHYISTNTTKKIPIILF-I-YILLMNMLH**KV**AVQRP-GQIPPLLNQLLKDQNVTLGFILKGLQGPAGFDGIPGAPGVQGPIGPPGYPGEMGPMGPPGL

110 120 130 140 150 160 170 180 190 200

....|....|....|....|....|....|....|....|....|....|....|....|....|....|....|....|....|....|....|....|

Polypodium_hydriforme MGPMGPPGLQGNPGQDGWPGAPGAPGMTGAPGSSGMPGPPGLPGLQGAPGAPGPTAIRY-NGTVKCEEDTAWLRCGEYKRISIISAFWGRRNFALCTEHT

Sphaeromyxa zaharoni KGDKGETGYPGKPGMDGWIGPPGSPGFPGEPGNSGPEGQAGPPGIPGEPGPPGLSSIR**Y**-**N**GTVKCEEDTAWLRCGEFKRISIVSVFWGRRSLAVCAEHT

Enteromyxum leei RGFTGEPGVAGEPGRDGLDGIQGPPGIPGDPGPAGMSGPPGPPGTPGTINGVVETRFNI**PN**ITLKCEEDTAWLKCGEYKRISVKSVFWGRRDFEKCAENN

Kudoa iwatai RGFPGEPGVPGEPGRDGNDGYPGAPGFPGEPGPSGMPGPPGPPGLPGDTPPFLPIVL**RN**-TTIIKCEEDTAWLKCVDYKKISIKSVFWGRRNFDICSENS

210 220 230 240 250 260 270

....|....|....|....|....|....|....|....|....|....|....|....|....|....|....|...

Polypodium_hydriforme GNLNSKKYCPTQPLFLTKVKDACEGTTICEIRCTKFFFHDKTCPDVYKYLEVYYKCIEVINGHEVVNEDNLLSANFAG

Sphaeromyxa zaharoni GDLYTDKFCPTDPMFLTKVKDTCEGTTMCEIRCTKTFFNDNHCPEIYKYLEVYYKCIEVINGHEVVNEENVLSGNMFG

Enteromyxum leei GNLFVDKYCPTQPLFLAKVKDACEGTTMCEIRCTKLFFNDKSCPDVYKYAEIDYDCVEIINGHEVVNNRHIIVE----

Kudoa iwatai GNLVTDKYCPTNPLFLAKVKDACDGTTMCEIRCTKLFFNDKTCPDVYKYAEIDYKCVEVINGHEVVNNERNVMGEI--

**Fig. S3.** Myxozoan and *Polypodium* nematogalectin-related. The sequence alignment was performed using Mafft and corrected manually. Nematogalectin C proteins contain a signal peptide (green-shaded areas) at the N terminus. A collagen domain made of repeats of tripeptide GlyXY (red-shaded areas) in the center and a sugar-binding galectin domain (cyan-shaded areas) at the C-terminus. Residues that span two exons (either a single residue spanning the exon-exon junction or two residues on each terminus of an exon), when intron positions are known, are indicated in gray.

10 20 30 40 50 60 70 80 90 100

....|....|....|....|....|....|....|....|....|....|....|....|....|....|....|....|....|....|....|....|

Polypodium hydriforme C MAVRIETISYLTYVLLIAGQLSQIYSTISDNDAVMWNLMGIDSPESEEEVDFSTGAKITNLSSEAGSVLLPPPSLTTSTLVNTTIAAADLAEAAEDSGDE

Enteromyxum leei C ----------------------------------------------------------------------------------------------------

Kudoa iwatai C ----------------------------------------------------------------------------------------------------

Sphaeromyxa zaharoni C ----------------------------------------------------------------------------------------------------

110 120 130 140 150 160 170 180 190 200

....|....|....|....|....|....|....|....|....|....|....|....|....|....|....|....|....|....|....|....|

Polypodium hydriforme C ETIQSGDFPSGDAASGDEAMAVEEEEQPGVFVDQAISPSLAKNMPFHTTRRLDDVSLGGGPIQVSAVVPSPADTIVTNTQRANASVLVQVVKNNAPANAT

Enteromyxum leei C ----------------------------------------------------------------------------------------------------

Kudoa iwatai C ----------------------------------------------------------------------------------------------------

Sphaeromyxa zaharoni C ----------------------------------------------------------------------------------------------------

210 220 230 240 250 260 270 280 290 300

....|....|....|....|....|....|....|....|....|....|....|....|....|....|....|....|....|....|....|....|

Polypodium hydriforme C SDAPSGPAVSALLDALAAYQANFTDPANANSTGNETQDTLNVDLPEY--PSLRNTSAISNSIATESAHKESLAPTIGKSAKTEKPEQNILPTVQN-----

Enteromyxum leei C ----------------------------------------------------------------------------------------------------

Kudoa iwatai C ----------------------------------------------------------------------------------------------------

Sphaeromyxa zaharoni C ----------------------------------------------------------------------------------------------------

310 320 330 340 350 360 370 380 390 400

....|....|....|....|....|....|....|....|....|....|....|....|....|....|....|....|....|....|....|....|

Polypodium hydriforme C ---ISQETTTPV---STSTQSSPLNNRTEEASPQVNSTGGLNDI--------LTSLMSEQADNKEDSGLTSSDAIMENLLMSVMSNDTTVKEELPGSDGD

Enteromyxum leei C ----------------------------------------------------------------------------------------------------

Kudoa iwatai C --------------------------------------------MLLLIKIRIYTANNLNTNNITSTNTLSYVEPILPSYSTKDVLLPYDSNATDTYNEN

Sphaeromyxa zaharoni C ----------------------------------------------------------------------------------------------------

410 420 430 440 450 460 470 480 490 500

....|....|....|....|....|....|....|....|....|....|....|....|....|....|....|....|....|....|....|....|

Polypodium hydriforme C VDMLNIDPSQSFLAEGSSDDNEGSAGVDGSSGGDSIIEMGSGEDTESSSIPLNLMEELAGTMGIVQPGKNTSVTEIIHIPGSLVPTSLEGTNSSGSVQGT

Enteromyxum leei C ----------------------------------------------------------------------------------------------------

Kudoa iwatai C DVILDNLMEVNNNLSKHVYSTLNNSTESL**A**NMSTLSNMKNDTPPVNNVREIVKKEHALLDQIQTNDTLPQNIFSKSNIKEISPKSYNESSHKEISFYNKS

Sphaeromyxa zaharoni C ----------------------------------------------------------------------------------------------------

510 520 530 540 550 560 570 580 590 600

....|....|....|....|....|....|....|....|....|....|....|....|....|....|....|....|....|....|....|....|

Polypodium hydriforme C DTSYPNATISVSVNTSSDSIKNNDDSRPFSGNDDNLTEERSSTAQILASADEIQYSKTKSSGSANGKPKTLEKTGAKKDILESNRGGSSYDSESDDDFNS

Enteromyxum leei C ----------------------------------------------------------------------------------------------------

Kudoa iwatai C VPSGSKMRDIPHPLVIANATLLLTPNNTNVLSLNNNSIVGLLKKLLFKPINNHSL**V**TTTLSNNKTVVHHTKNKTYKIAPVIF**N**SLNKLLAHKNISYTDDI

Sphaeromyxa zaharoni C MFSLKRTFEQITG**DD**QQIGSFLKNKGNQNEYLQEDKPSLTTRLVVEGIKSDMHFVNENATRNRIKIRSLDDETTEPGTYDNIEASSSAEQYIDSDGKNYN

610 620 630 640 650 660 670 680 690 700

....|....|....|....|....|....|....|....|....|....|....|....|....|....|....|....|....|....|....|....|

Polypodium hydriforme C DFIEVSGDSDGSDEDEEPDEREEEEDNKDSDKSQDQDISGLS------YEDRLILSQMLDQNLVLFYTSMS-----GNPGVVGKTGKKGKRGFAGMPGPP

Enteromyxum leei C ----------------------------------------PG------INIDDLTQKALDMNLFLFYAPISLLGWSGNIGNAGPAGETGEKGEIGVPGKP

Kudoa iwatai C HNNR**IE**RAHSLKSSLILPNISKNIKKSYITDRSNDEILHSTTPTP**LK**LIELENEVKKVLKMNLFTFFAPISLLGLTGPPGLPGEPGPPGFPGNVGIPGNP

Sphaeromyxa zaharoni C NSEESVRMENNNGQSEFGDLSEGHKKSKLAKKS**KK**KSKRE------KQEKIDPLTKFVLNMPLYTFAAPADILMLIGPPGARGKTGPPGSMGVIGFPGRP

710 720 730 740 750 760 770 780 790 800

....|....|....|....|....|....|....|....|....|....|....|....|....|....|....|....|....|....|....|....|

Polypodium hydriforme C GHRGEPGPPGFPGRQGSVGPKGPRGPAGQPGLKGPQGDPGEVGDPGIPGP---PGMRGLPGPMGDFGEGPVLP-NCSIVCEGEKDWIECREYETVKIQKV

Enteromyxum leei C GRRGIMGE---AGVT**G**PPGVKGDVGIPGAPGFVGDKGEPGSIGIPGNMGPKGETGSIGLPGVPGLSSDYIQA**Q**-**N**CTHACGNEKVWIDCKKYEVININSA

Kudoa iwatai C GFPGRVGI---AGRQ**G**PMGPQGVIGNPGEPGDQGIKGPPGDPGPPGIVGQTGNPGLEGPPGEPGTPADEYLI**P**-**N**CTYICGNDKYWLECKQFEVINVITA

Sphaeromyxa zaharoni C GSPGQDGP---IGER**G**PLGPKGPTGSPGVPGQRGEQGLSGGVGSPGDIGDQGQDGFIGPPGSPGQVGEEAQIPV**NC**SYVCQNEKIWLQCREYEVIHIIKA

810 820 830 840 850 860 870 880 890 900

....|....|....|....|....|....|....|....|....|....|....|....|....|....|....|....|....|....|....|....|

Polypodium hydriforme C FWGRDDFD-ICTKAPLGLRTDAYCDGDPENAFKKVEDQCRGKRACEVVASNIFFDDSSCGNVFKYLKVCYECVLETTD--SGELLGGSKRRRRRREGERH

Enteromyxum leei C FWGRDENNQICLNAPQGLSTDRSCSKDSDLVKRKVSDQCTGKSACEVVASNIFFDDNDNSDVYKYLEVCYQCDPDEIGLSANEVLNYSIKR---------

Kudoa iwatai C FWGRENDF-MCPNYPNGLTIQKKCSLDPDIAWKKVSDQCNNKNACELIATNLFFDDNQHDDVYKYAKVCYECAIDE----ANILKGYDKKRRSTKT----

Sphaeromyxa zaharoni C YYGRNDLD-ICSDAPDGMKSNILCEGDQNRIYLKVVDQCQNREACELVASNIFFDDNSCPDVYKFLTICHECIPDDSDPMVNTLLNMAKSRKKRNSLRNG

....|...

Polypodium hydriforme C RRLAHP--

Enteromyxum leei C --------

Kudoa iwatai C --------

Sphaeromyxa zaharoni C ITLISKEK

**Fig. S4.** Myxozoan and *Polypodium* nematogalectin C. The sequence alignment was performed using Mafft and corrected manually. Nematogalectin C proteins contain a signal peptide (green-shaded areas) at the N terminus. A collagen domain made of repeats of tripeptide GlyXY (red-shaded areas) in the center and a sugar-binding galectin domain (cyan-shaded areas) at the C-terminus. Residues that span two exons (either a single residue spanning the exon-exon junction or two residues on each terminus of an exon), when intron positions are known, are indicated in gray.
